# *Adhatoda vasica* and *Tinospora cordifolia* extracts ameliorate clinical and molecular markers in mild COVID-19 patients: a randomized open-label three-armed study

**DOI:** 10.1186/s40001-023-01507-7

**Published:** 2023-12-04

**Authors:** Mukta Verma, Neha Rawat, Ritu Rani, Manju Singh, Aditi Choudhary, Sarfaraz Abbasi, Manish Kumar, Sachin Kumar, Ankur Tanwar, Bishnu Raman Misir, Sangeeta Khanna, Anurag Agrawal, Mohammed Faruq, Shalini Rai, Richa Tripathi, Anil Kumar, Mukta Pujani, Meera Bhojani, Anil Kumar Pandey, Tanuja Nesari, Bhavana Prasher

**Affiliations:** 1 Centre of Excellence for Applied Development of Ayurveda Prakriti and Genomics, CSIR-Institute of Genomics & Integrative Biology, Delhi, India; 2 CSIR-Institute of Genomics & Integrative Biology, Delhi, India; 3 All India Institute of Ayurveda, New Delhi, India; 4 ESIC Medical College and Hospital, Faridabad, Haryana India; 5 Trivedi School of Biosciences, Ashoka University, Sonipat, Haryana India

**Keywords:** COVID-19, Clinical trial, *Adhatoda vasica*, *Tinospora cordifolia*, Anti-hypoxic, Anti-inflammatory, Immunomodulatory

## Abstract

**Background:**

SARS-CoV-2 infections caused mild-to-moderate illness. However, a sizable portion of infected people experience a rapid progression of hyper-inflammatory and hypoxic respiratory illness that necessitates an effective and safer remedy to combat COVID-19.

**Methods:**

A total of 150 COVID-19-positive patients with no to mild symptoms, between the age groups 19–65 years were enrolled in this randomized, open-labeled three-armed clinical trial. Among them, 136 patients completed the study with RT-PCR negative reports. The patients received herbal drugs orally (Group A (*Adhatoda vasica*; AV; 500 mg; *n* = 50); Group B (*Tinospora cordifolia*; TC; 500 mg; *n* = 43), and Group C (AV + TC; 250 mg each; *n* = 43)) for 14 days. Clinical symptoms, vital parameters, and viral clearance were taken as primary outcomes, and biochemical, hematological parameters, cytokines, and biomarkers were evaluated at three time points as secondary outcomes.

**Results:**

We found that the mean viral clearance time was 13.92 days (95% confidence interval [CI] 12.85–14.99) in Group A, 13.44 days (95% confidence interval [CI] 12.14–14.74) in Group B, and 11.86 days (95% confidence interval [CI] 10.62–13.11) days in Group C. Over a period of 14 days, the mean temperature in Groups A, and B significantly decreased linearly. In Group A, during the trial period, eosinophils, and PT/INR increased significantly, while monocytes, SGOT, globulin, serum ferritin, and HIF-1α, a marker of hypoxia reduced significantly. On the other hand, in Group B hsCRP decreased at mid-treatment. Eosinophil levels increased in Group C during the treatment, while MCP-3 levels were significantly reduced.

**Conclusions:**

All the patients of the three-armed interventions recovered from COVID-19 and none of them reported any adverse effects from the drugs. Group C patients (AV + TC) resulted in a quicker viral clearance as compared to the other two groups. We provide the first clinical report of AV herbal extract acting as a modifier of HIF-1α in COVID-19 patients along with a reduction in levels of ferritin, VEGF, and PT/INR as the markers of hypoxia, inflammation, and thrombosis highlighting the potential use in progression stages, whereas the TC group showed immunomodulatory effects.

*Trial registration* Clinical Trials Database -India (ICMR-NIMS), CTRI/2020/09/028043. Registered 24th September 2020, https://www.ctri.nic.in/Clinicaltrials/pdf_generate.php?trialid=47443&EncHid=&modid=&compid=%27,%2747443det%27

**Graphical Abstract:**

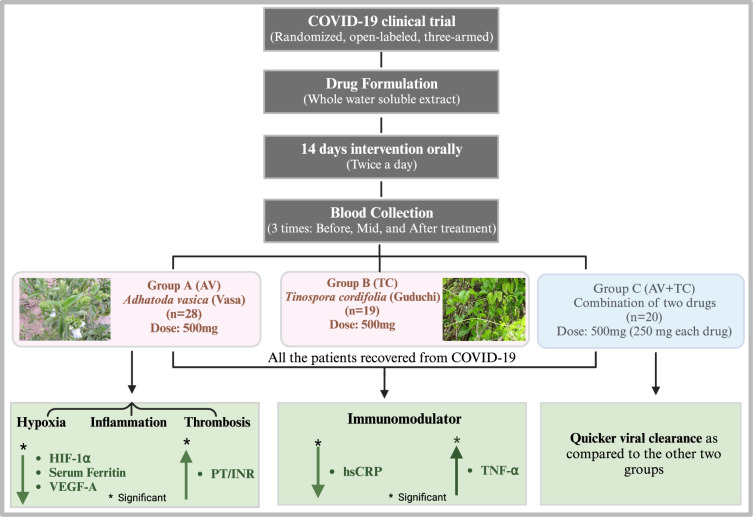

**Supplementary Information:**

The online version contains supplementary material available at 10.1186/s40001-023-01507-7.

## Background

The severe acute respiratory syndrome coronavirus 2 (SARS-CoV-2) that causes the coronavirus disease (COVID-19) has emerged as a global pandemic [1–3]. Most SARS-CoV-2 patients manifested mild-to-severe respiratory symptoms between 2 and 14 days of exposure, along with additional symptoms such as fever, coughing, and shortness of breath. In severely ill COVID-19 patients, the dysregulated, and uncontrolled production of pro-inflammatory cytokines such as higher levels of interleukins-6 (IL-6), Tumor necrosis factor α (TNF-α), ferritin, and C-reactive protein (CRP) leading to hypoxic respiratory failure are the major contributor to acute respiratory distress syndrome (ARDS) and are linked to a severe deterioration in health [4–6]. Earlier research has found an association between elevated circulating D-dimer levels pointing to a specific coagulation pathology linked with COVID-19 [7, 8]. This finding is also supported by other studies in which COVID-19 patients show substantial extracellular fibrin deposition, and fibrin thrombi within dilated small arteries, and capillaries [9]. The link between hypoxia and coagulation is well documented, and studies have shown that HIF-1α, and vWF play key roles in the development of thrombosis, and bleeding susceptibility in response to hypoxia [10]. To eliminate the virus in a non-severe state or prevent development to a severe state during incubation, a specific humoral, and cell-mediated adaptive immune response is essential. Thus, in such states, measures required to increase immunological responses become significant [11, 12]. Since corticosteroids have been shown to improve clinical outcomes in COVID-19 patients with severe inflammation, and pneumonia, therefore, it is possible that other, more targeted immunomodulatory, anti-hypoxic, and anti-thrombotic medications will have similar effects [13]. Thus, recommended supportive therapies for acute hypoxia-induced respiratory failure were included in COVID-19 treatment [6].

*Adhatoda vasica* (AV)* (*Nees.) also known as *Vasa*/*Adulsa* in Ayurveda has *pitta kapha* balancing activity with bitter taste, and cooling effect. It is useful in the treatment of chronic bronchitis, asthma, colds, cough, fever, dyspnea, phthisis, blood disorders, jaundice, and diarrhea [14–16]. In earlier studies, we have demonstrated that water-soluble extract of AV significantly decreases the severity of airway inflammation caused by an enhanced hypoxic response in asthmatic mice who are resistant to other treatments [17]. We particularly observed inhibition of HIF-1α levels (hypoxia-inducible factor-1Alpha) in vivo, which was further confirmed in in vitro cellular model of hypoxia. Additionally, AV prevented hypoxia-induced mitochondrial dysfunction and reversed oxidative phosphorylation and ATP synthesis in lung epithelial cell lines. In severe COVID-19 cases, altered bioenergetic profiles of monocytes in response to elevated HIF-1α are a relevant molecular marker. Further, AV attenuates the increased levels of TGF-β1, IL-6, and HIF-1α in the mouse models of pulmonary fibrosis, and sepsis. AV treatment rescues the PHD2 siRNA-induced hypoxia, inflammation, and thrombosis phenotypes in mice. In addition to the potential to modify the host response, AV was also shown to inhibit the SARS-CoV2 virus load in infected Vero cell culture [18]. In silico studies have shown that along with other chemical constituents of AV, the alkaloid compound vasicine has a very good binding affinity against SARS-CoV-2 viral proteins such as 3CL-pro and RdRp, and host receptors such as angiotensin converting enzyme (ACE-2) and TMPRSS2 [18, 19].

Similarly, the second herb, *Tinospora cordifolia *(Thunb.) Miers (TC), also called *Guduchi* in Ayurveda, is known for its diverse applications in various diseases. *Guduchi* has *Rasayana* effect/homeostatic potential with all three *Dosha* balancing activities. It also has a bitter and astringent taste with hot potency. Numerous researches on TC have demonstrated its potent antioxidant, hepatoprotective, cardiovascular protective, anti-inflammatory, antipyretic, thrombolytic, anti-microbial, and anti-cancer effects [20, 21]. It has been demonstrated that TC modulates the activity of a variety of immune response cells, which is either directly or indirectly essential for enhancing immunity. According to reports, TC stimulates macrophages' phagocytic activity [22]. It also activates the cytotoxic T cells and promotes the B cell differentiation [23]. All these effects are relevant in producing the anti-viral impact, and eradicating viruses such as SARS-CoV2 in the early phase of infection [20, 24]. M/o AYUSH national clinical management protocol based on Ayurveda and Yoga also includes *Guduchi ghan* tablet for managing asymptomatic and mild cases of COVID-19 [25]. Additionally, virtual screening and molecular docking studies of the phytochemical components of TC can reduce or prevent SARS-CoV-2 entrance and consequent infectivity by blocking the interaction between the receptor binding domain (RBD), and ACE2 [26]. Compounds like xanosporic acid, tinosponone, cardiofolioside B, berberine, and tembetarine of TC were identified as potential lead compounds to combat SARS-CoV-2 [27].

In the present study, the clinical efficacy of AV, TC, and a combination of both in terms of primary outcomes such as management of COVID-19 symptoms, prevention from progression to severe stages and viral clearance was examined. Additionally, we also studied the effects of treatment on inflammatory cytokines as well as the makers of hypoxia and thrombosis. To evaluate the systemic effects of the disease, as well as that of trial medication, parameters such as CBC, LFT, KFT, and lipid profile were also tested as secondary outcomes. Here we report the results of a randomized, preclinical lead-based, open-labeled three-armed clinical trial aimed at evaluating the direct effects of AV (*Vasa Ghan*) and TC (*Guduchi Ghan*) individually, and their combination’s effect on SARS-CoV2-positive patients with no or mild symptoms. We observed overall these herbal formulations exhibited no adverse effects, all the patients recovered clinically and did not progress to a severe form of the disease. In addition to other anti-inflammatory and anti-thrombotic markers, preclinical observations of *Adhatoda vasica* on HIF-1α inhibition were also observed. To the best of our knowledge, this is the first investigation into the therapeutic effects of the herbal formulation administered standalone in COVID-19 positive cases with regular monitoring of the symptoms as well as viral clearance and blood parameters done at three time points. This study suggests the inclusion of AV and TC as standard care for COVID-19 positive patients.

## Methods

### Study design, participants, and ethics

A three-armed, open-labeled, randomized clinical trial was undertaken from November 2020 to September 2021. A total of 150 patients were recruited at the screening OPD and COVID health centre of All India Institute of Ayurveda (AIIA), New Delhi and ARI (acute respiratory infection) OPD and Corona Ward, ESIC Medical College and Hospital, Faridabad, Haryana. The inclusion criteria were (1) diagnosis of COVID-19 positive based on RAT and RT-PCR results; (2) age 19–65 years; (3) presenting with no or mild symptoms (SpO_2_ ≥ 93% on room air); (4) willingness to participate in the study with an ability to take the drug orally; (5) written informed consent by the patient. The exclusion criteria were (1) COVID-19 patients with symptoms classified as moderate, severe, or critical decided on the basis of parameters such as SpO_2_ < 90% on room air, respiratory distress at room ambience (≥ 30 beats per minute), any of the known COVID-19 complications, requirement of oxygen for more than one hour [28]; (2) individuals with uncontrolled, unstable co-morbidities; (3) immuno-compromised individuals; (4) patients already taking any other kind of Ayurveda treatment or any other intervention from alternative and complementary medicinal systems such as homeopathy, Unani, and Siddha for management of COVID-19; (5) pregnant and lactating women; (6) patients with an altered mental state; (7) participation in any other clinical trial with therapeutic intervention. The case record form was created for documentation of clinical assessment, and monitoring at baseline, during the trial period for subsequent analysis.

The 150 random numbers were generated using 3-digit random numbers tables and allocation to 3 groups was done: Group A (even number), Group B (odd number) and Group C (number multiple of 3). As per this, 52 patients in Group A, 51 patients in Group B and 47 patients in Group C were enrolled in the study. This study was carried out in accordance with the recommendations of the Indian Council of Medical Research, India guidelines for biomedical research, with written informed consent from all subjects. The study protocol was approved by the Institutional Human Ethics Committee of CSIR-Institute of Genomics and Integrative Biology (IGIB), New Delhi, All India Institute of Ayurveda (AIIA), New Delhi and ESIC Medical College and Hospital, Faridabad, Haryana, India. The study was registered in the Clinical Trials Database with Registration no. CTRI/2020/09/028043. The trial drug capsules of extracts of *Adhatoda vasica (Nees.)* and *Tinospora cordifolia (Thunb.) Miers* were procured from Good Manufacturing Practice (GMP) certified manufacturer following the quality control and analysis methods as per pharmacopeial standards. Same colored capsules filled in identical containers for ***Vasa* Ghan** (a whole water-soluble extract): 500 mg BD, ***Guduchi* Ghan** (a whole water-soluble extract): 500 mg BD and ***Vasa Guduchi* Ghan** (a whole water-soluble extract): 500 mg BD (250 mg each of *Vasa* and *Guduchi*) were dispensed to patients for taking twice a day for 14 days daily.

At the time of enrollment, patients were provided information on how to record their vitals and report their clinical symptoms daily such as temperature, pulse, blood pressure (BP), respiratory rate (RR) and oxygen saturation levels. They were provided pulse oximeters to facilitate accurate reporting of SpO_2_. They were also informed about the protocol for mid and end study blood sample collection and RT-PCR testing.

**Fig. 1:**
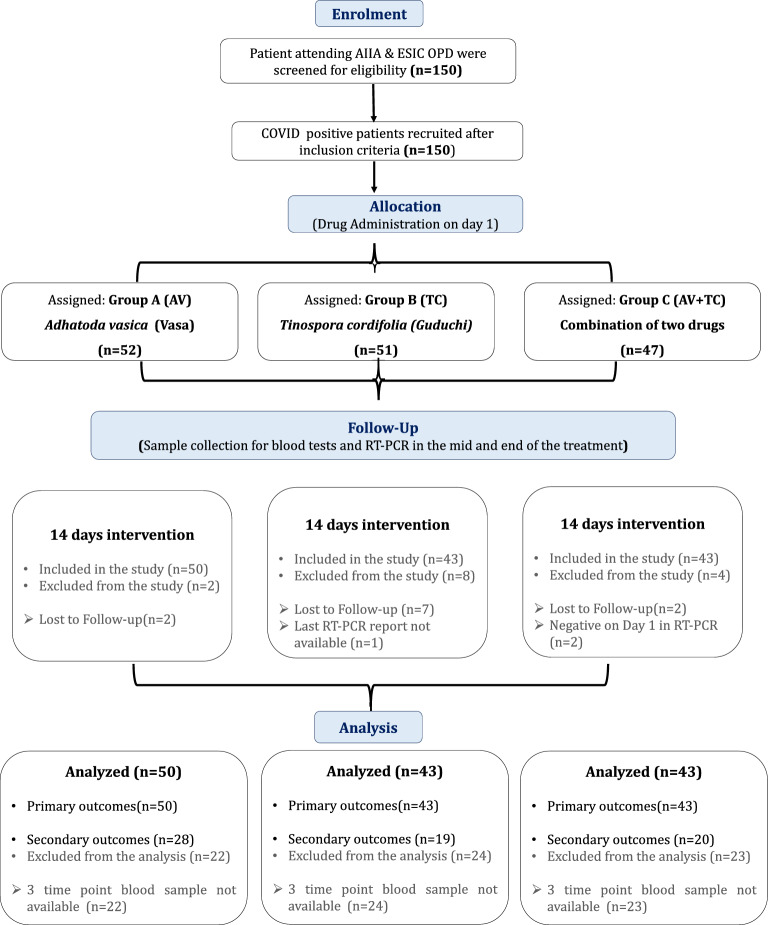
Recruitment and randomization of the patients. 150 COVID-19 positive patients were enrolled in the clinical trial. We randomized 150 patients who met the inclusion criteria into Group A (*n* = 52), Group B (*n* = 51), and Group C (*n* = 47). 50, 43, and 43 patients in Group A, B, and C, respectively, completed the study. Among 150 patients, 14 patients (two, eight, and four from Group A, B, and C, respectively) were lost to follow-up. One patient from Group B completed the intervention and follow-up, but was not included in analysis for unavailability of post-treatment sample. The analysis of primary outcome was done on 136 patients, 50 in Group A, and 43 in each Group B, and C. The analysis of secondary outcome was done on a total of 67 patients who gave their blood samples at three timepoints Group A (*n* = 28), Group B (*n* = 19), and Group C (*n* = 20)

After enrollment in the intervention, 14 patients (two, eight, and four from AV, TC, and AV + TC, respectively) were lost to follow-up. Among these, eleven individuals did not provide any information about medication taken/ not and no follow-up/tests could be done after enrollment. Two patients were enrolled based on RAT, however they turned negative on RT-PCR, hence did not initiate the treatment. One patient took intervention and provided clinical follow-up for the recovery of symptoms but did not come for final blood/RT-PCR tests, hence the data were not included in the final analysis. A total of 136 patients completed the study with RT-PCR negative, out of which 67 subjects gave their blood samples at the three time points (Fig. 1).

### Outcomes

#### Primary outcomes

The amount of time taken for RT-PCR to turn negative served as a primary outcome of viral clearance. Clinical symptoms such as fever, cough, difficulty breathing, dyspnea, sore throat, headache, joint pain, fatigue, running nose, nasal congestion, loss of sense of smell, loss of sense of taste, myalgia, nausea, vomiting, and diarrhea surveilled two times a day, i.e., morning and evening along with other vital parameters like temperature, heart rate (HR), blood pressure (BP), respiratory rate (RR), and oxygen saturation (SpO_2_) for 14 days**.** For vital parameter analysis, the morning and evening values for each day of every individual were averaged and then compared them across individuals.

#### Secondary outcomes

Blood tests planned to be conducted in the study were mainly of two types: (1) for assessment, monitoring the disease progression markers (PT-INR, Hs CRP, D-dimer, ferritin, cytokines, and other molecular markers) and observing the effect of trial medicines on them; (2) for identifying systemic effects of the disease as well as the effect of trial medication on the general safety of these parameters such as CBC, LFT, KFT, lipid profile. To evaluate these blood parameters, 16 ml venous blood was collected at three time points before the start of treatment, midway through treatment, and at the end of treatment. Hematological parameters, CBC, and ESR were analyzed on a 6-part hematology autoanalyzer (Sysmex XN 1000), and ESR analyzer (Vesmatic 80), respectively. Coagulation parameters (PT/INR/D-dimer) were analyzed by a fully automated coagulation analyzer (STAGO startmax). Biochemical parameters (LFT, KFT, lipid profile, ferritin, and hsCRP) were analyzed on a fully automated analyzer (Vitros XT 7600).

Further serum samples were isolated and stored to measure 15 pro-inflammatory and anti-inflammatory cytokines (IFN-α2, IFN-γ, IL-1β, IL-1RA, IL-4, IL-6, IL-7, IL-8, IL-13, IL-18, IP-10, MCP-1, MCP-3, TNF-α, and VEGFA) levels through multiplexing (MILLIPLEX® MAP KIT; Cat.#HCYTA-60K), and the plate was scanned using MAGPIX with xPONENT software and measuring biomarkers such as HIF-1α (ELISA kit-Elabscience®; E-EL-H6066), and vWF (ELISA kit-Elabscience®; E-EL-H2168) and the plate was scanned using Tecan i-control, 2.0.10.0. All the experiments were performed by following the manufacturer's protocols. 67 patients' data with three time points were considered for the analysis of secondary outcomes.

X-rays were also done for patients at the time of enrollment and at the end of the intervention period, to rule out any pulmonary involvement or progression during the course of disease in patients on treatment.

### Statistical analysis

The data processing and statistical analysis were performed using IBM Statistical Package for Social Sciences Software (SPSS) for Windows, version 26 and R software (v.4.1.2). The data distribution was examined for each parameter using the Shapiro–Wilk test (*p* > 0.05). The one-way ANOVA was used to examine normally distributed parameters, and the Friedman test was used for non-normally distributed parameters for time-point comparison. The paired t-test and Wilcoxon sign rank test were used to compare the time points in each group for pairwise comparison for time-point comparison. The Kruskal–Wallis test and Wilcoxon sign rank test were used for group-wise comparison. The Bonferroni post hoc correction was used for multiple comparison corrections. Repeated measure ANOVA was used to compare primary outcome parameters measured for 14 consecutive days. Time-to-negativity analysis was reported with a Kaplan–Meier survival plot. *p* < 0.05 was considered statistically significant.

## Results

A total of 150 patients diagnosed with COVID-19 using a RAT or RT-PCR positive test were enrolled among them 101 were males and 49 females with mean age 38.59 ± 11.74 years. Fourteen patients were considered to drop out of the trial. Among 136 patients, 50 patients were in Group A (*Vasa* treated group), 43 patients were in each Group B (*Guduchi* treated group), and in Group C (*Vasa* + *Guduchi* treated group) (Fig. 1).

All the groups had similar demographic and illness characteristics (Table 1). The proportions of patients who complained of fever were 60% in Group A, 55% in Group B, and 53.49% in Group C at the time of enrollment, and with cough in 54%, 57.5%, and 37.21% in Group A, B, and C, respectively (Table 1). There was an improvement in fever, sore throat, headache, loss of smell, and loss of taste in all the groups after the treatment.

**Table 1:** Baseline demographics and clinical features of COVID-19 patients in each group

Demographic and clinical features	Group A (*n* = 50)	Group B (*n* = 43)	Group C (*n* = 43)
Age, mean ± SD, years	39.90 ± 11.34	38.41 ± 11.38	39.06 ± 12.71
Gender, M/F	33/17	28/15	30/13
Time between first diagnosis and enrollment, median (IQR), days	1 (0.25–2)	1 (0–2)	1 (1–2)
Fever, no. (%)	30 (60)	24 (55.81)	23 (53.49)
Cough, no. (%)	27 (54)	24 (55.81)	16 (37.21)
Difficulty in breathing, no. (%)	6 (12)	5 (11.63)	3 (6.98)
Dyspnea, no. (%)	2 (4)	3 (6.98)	2 (4.65)
Sore throat, no. (%)	14 (28)	15 (34.88)	18 (41.86)
Headache, no. (%)	11 (22)	13 (30.23)	14 (32.56)
Joint pain, no. (%)	4 (8)	6 (13.95)	3 (6.98)
Fatigue, no. (%)	8 (16)	9 (20.93)	5 (11.63)
Running nose, no. (%)	3 (6)	3 (6.98)	0 (0)
Nasal congestion, no. (%)	1 (2)	3 (6.98)	3 (6.98)
Loss_Smell, no. (%)	14 (28)	13 (30.23)	8 (18.6)
Loss_Taste, no. (%)	11 (22)	13 (30.23)	6 (13.95)
Diarrhea, no. (%)	2 (4)	1 (2.33)	0 (0)
Vomiting, no. (%)	1 (2)	1 (2.33)	1 (2.33)

n, number of patients; no., number; %, percent; SD, standard deviation

### Viral clearance

**Fig. 2:**
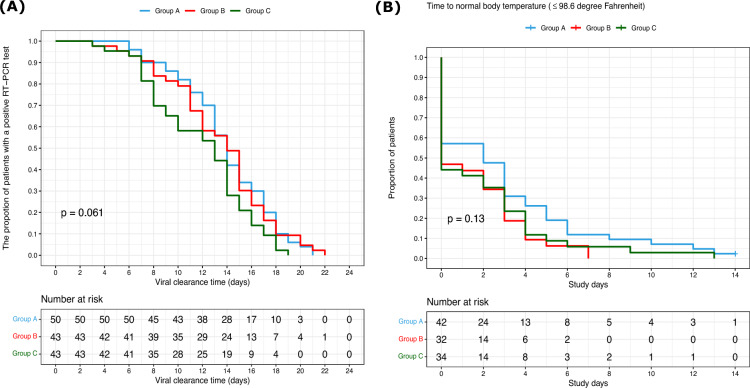
Kaplan–Meier survival analysis of the proportion of patients: **A** positive RT-PCR test and **B** time to normal body temperature

Patients were mostly home quarantined and opted to come for follow-up RT-PCR at their convenience. Hence this is more indicative of the confirmation date rather than the actual time to negativity which could be earlier. The mean time taken to become COVID-19 negative was 13.92 days (95% confidence interval [CI] 12.85–14.99) in Group A (*n* = 50), 13.44 days (95% confidence interval [CI] 12.14–14.74) in Group B (*n* = 43), and 11.86 days (95% confidence interval [CI] 10.62–13.11) in the Group C (*n* = 43). The difference of 2.06 days was the time reduced by Group C in comparison to Group A alone, and that was significant (*p* = 0.048). From the results, it was also clear that Group A and B both had taken nearly equal time to COVID-19 negativity (Fig. 2A). Using Kaplan–Meier survival analysis, the percent of patients with a positive RT-PCR test was 80% in Group C, and 90% in Group A and B on the 7th day of the trial period. It was 20% in Group C, 30% in Group B and 36% in Group A on the 15th day of the trial period.

### Time to normal body temperature

At the time of recruitment, 40% of patients had a temperature ≤ 98.6°F (normal body temperature) in Group A and < 50% of patients in Group B and C. Patients had higher body temperature recovered with the median day of 4 (95% confidence interval [CI] 3.86–6.72), 3 (95% confidence interval [CI] 2.66–4.41), 4 (95% confidence interval [CI] 2.86–5.94) in Group A, B, and C, respectively. On the 7th day, nearly 90% of patients had a temperature ≤ 98.6 °F in Group B, C, and A (Fig. 2B).

### Primary clinical outcome

**Fig. 3:**
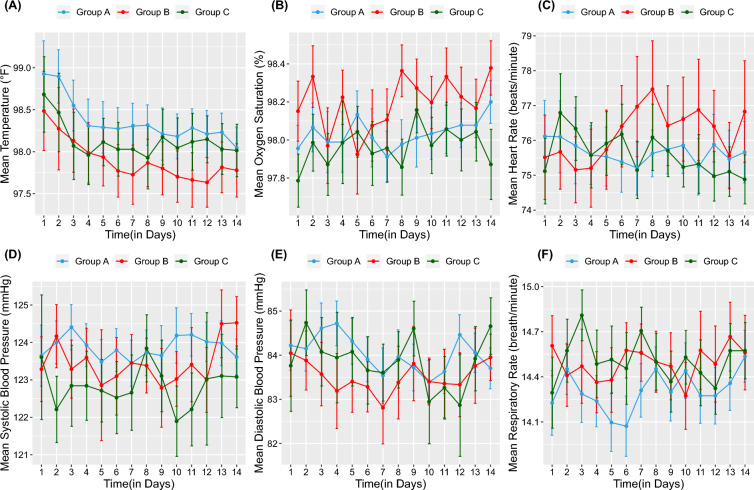
Trend in mean clinical symptoms in three study Groups over 14 days of observations: **A** temperature (℉), **B** oxygen saturation (%), **C** heart rate (bpm), **D** systolic blood pressure (mmHg), **E** diastolic blood pressure (mmHg), **F** respiratory rate (breath\min). ℉, degree Fahrenheit; bpm, beats per minutes; mmHg, millimeters of mercury; %, percent

The average morning and evening data of each individual were assessed to compare the trend in their vital parameters for 14 consecutive days. There was a significant decline in mean temperature over 14 days from 98.92 ± 1.28 to 98.04 ± .80 in Group A; *n* = 42, from 98.49 ± 1.33 to 97.7 ± .89 in Group B; *n* = 32, and from 98.68 ± 1.29 to 98.01 ± .89 in Group C; *n* = 33 (Fig. 3A). However, between-group variations were insignificant. Initially, on Day 1, the levels of oxygen saturation (SpO_2_) (Fig. 3B), heart rate (HR) (Fig. 3C), blood pressure (BP) (Fig. 3D, E), and respiratory rate (RR) (Fig. 3F) were within the normal range, and it remained the same during the trial period. At baseline mean SpO_2_ was 97.95 ± 0.81% in Group A (*n* = 45), 98.15 ± 0.91% in Group B (*n* = 33), and 97.79 ± 0.83% in Group C (*n* = 35), mean HR was 76.12 ± 6.76 bpm in Group A (*n* = 43), 75.52 ± 6.85 bpm in Group B (*n* = 32), and 75.12 ± 5.47 bpm in Group C (*n* = 34), mean systolic BP was 123.64 ± 4.24 mmHg in Group A (*n* = 27), 123.28 ± 3.95 mmHg in Group B (*n* = 21), and 123.60 ± 7.25 mmHg in Group C (*n* = 19), and mean diastolic BP was 84.22 ± 2.91 mmHg in Group A (*n* = 27), 84.04 ± 4.48 mmHg in Group B (*n* = 21), and 83.76 ± 4.51 mmHg in Group C (*n* = 19). Further mean RR was 14.23 ± 1.40 breath per minute in Group A (*n* = 42), 14.60 ± 1.15 breath per minute in Group B (*n* = 33), and 14.29 ± 1.37 breath per minute in Group C (*n* = 34) on Day 1. No significant differences or linearity in trend was observed in any of the three groups or between groups in these parameters.

### Secondary clinical outcome

#### Biochemical and hematological parameters

**Fig. 4:**
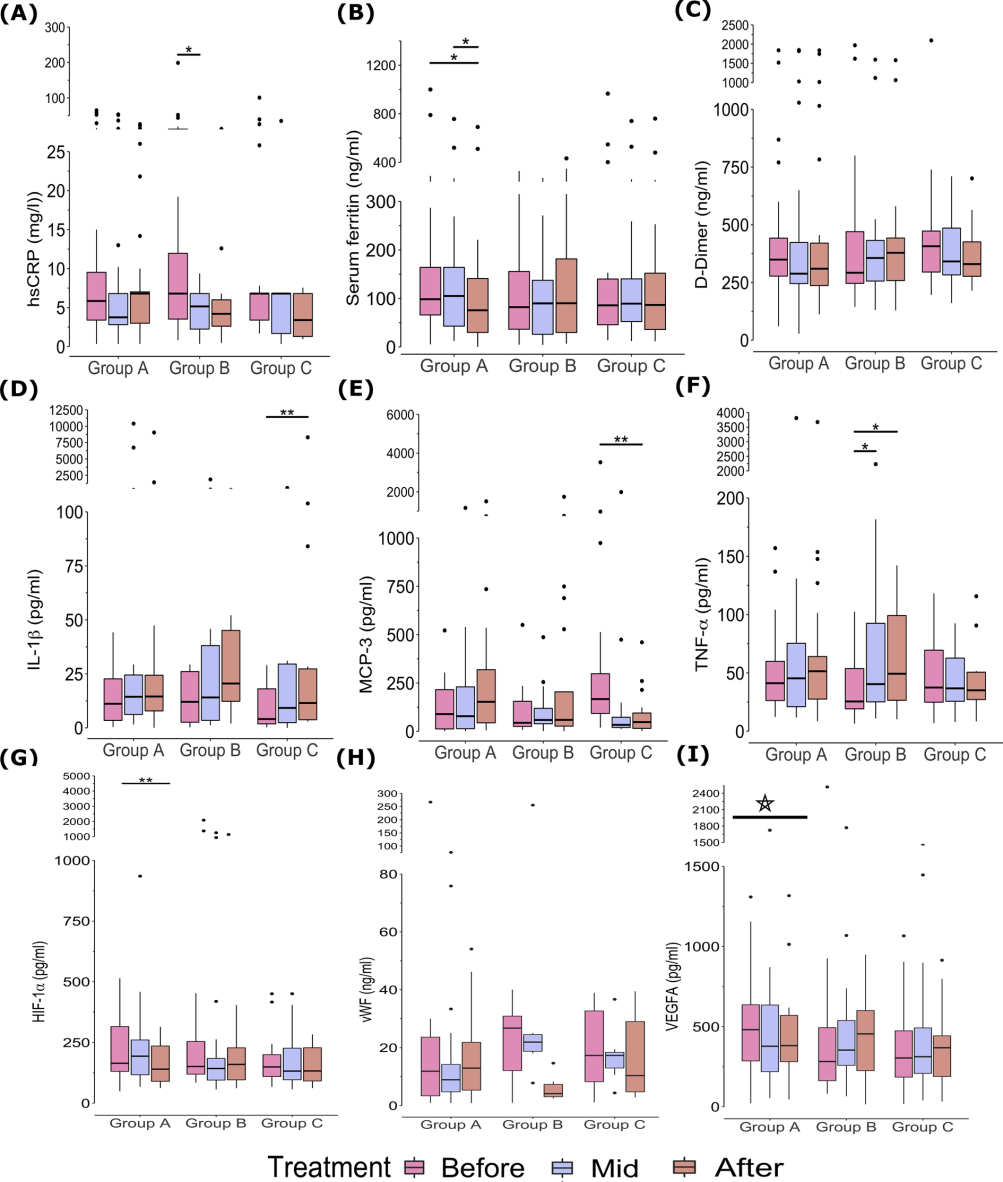
The levels of biochemicals and molecular markers: **A** hsCRP, **B** serum ferritin, **C** D-dimer, **D** IL-1β, **E** MCP-3, **F** TNF-α, **G** HIF-1α, **H** vWF, and **I** VEGFA at three timepoints in all the groups. HIF-1α, hypoxia-inducible factor-1 alpha; hsCRP, high-sensitivity C-reactive protein; ng/ml, IL, interleukin;, MCP-3, monocyte chemotactic protein-3; mg/ml, milligrams per millimeters; ng/ml, nanograms per milliliter; pg/ml, picograms per millimeters; TNF-α, tumor necrosis factor-alpha; VEGFA, vascular endothelial growth factor A; vWF, von Willebrand factor; star, In Group A Friedman,* p* = 0.013; * *p* < 0.05; ** *p* < 0.01; *** *p* < 0.001; **** *p* < 0.0001

The various hematological parameters such as CBC, ESR, and PT/INR were observed in the normal range and remained within the normal range even after treatment in all three groups. Although mild changes were observed in some parameters which were statistically significant after Bonferroni post hoc correction. In Group A, the percentage of eosinophil significantly increased (treated with *Vasa*) during treatment whereas monocyte decreased, this led to a slight increase in NMR from 6.57 ± 1.89 to 7.71 ± 2.14 (Additional file 1). Further, the PT/INR was increased from baseline to the end of the treatment although remained within the normal range. Considering liver function parameters, serum glutamic oxaloacetic transaminase (SGOT) was slightly higher than normal at baseline which decreased significantly at the end of the treatment period in Group A. Kidney function parameters were in the normal range at baseline and remained within range; slight decrease in globulin was observed. The lipid profile was in the normal range and remained unchanged (Additional file 1). Biochemical markers related to COVID-19 progression and severity such as serum ferritin levels were significantly decreased from baseline to end of the treatment (Table 2 and Fig. 4A, B). Levels of D-dimer decreased during the course of treatment, however, not statistically significant.

**Table 2:** Group-wise comparison of immunological and molecular markers at baseline (before treatment) and after treatment as well as timepoint-wise comparison within group

Sr. no	Parameters	Groups	Before treatment	Mid treatment	After treatment	P-value (time-point comparison in each group)	Kruskal P-value (group-wise comparison at baseline)	Kruskal P-value (group-wise comparison at after treatment)
1	IFN-α2 (pg/ml)	Group A	10.36 (5.38–17.52)	11.3 (5.7–23.81)	11.3 (7.88–20.1)	0.78	0.531	0.368
		Group B	8.65 (4.05–13.08)	7.88 (1.37–12.43)	7.88 (4.34–20.1)	0.37		
		Group C	11.3 (2.42–15.98)	6.15 (2.42–17.52)	6.15 (2.23–17.74)	0.3		
2	IFN-γ (pg/ml)	Group A	5.29 (2.43–13.88)	6.21 (1.86–13.88)	6.41 (1.82–10.83)	0.2	0.23	0.768
		Group B	1 (0.69–8.56)	9.39 (5.82–13.06)	11.37 (1–18.34)	0.06		
		Group C	7.76 (2.75–13.88)	6.81 (3.91–11.24)	5.35 (1–8.25)	0.88		
3	IL-1β (pg/ml)	Group A	11.15 (3.45–22.69)	14.4 (6.21–24.49)	14.51 (7.86–24.37)	0.09	0.314	0.978
		Group B	12.04 (2.62–26.07)	14.08 (3.48–38.09)	20.53 (12.3–45.08)	0.69		
		Group C	4.08 (1.88–18.07)	9.24 (2.41–29.54)	11.51 (3.77–27.31)	0.16,^##^		
4	IL-1Ra (pg/ml)	Group A	34.05 (17.74–64.02)	39.88 (14.57–68.21)	33 (24.66–76.63)	0.73	0.378	0.483
		Group B	22.33 (14.32–32.16)	37.28 (9.97–58.86)	41.07 (10.99–74.45)	0.53		
		Group C	28.74 (10.83–47.4)	28.24 (13.03–58.5)	39.62 (27.14–70.46)	0.14		
5	IL-4 (pg/ml)	Group A	0.48 (0.24–1.38)	0.24 (0.17–0.75)	0.3 (0.24–0.75)	0.15	0.568	0.688
		Group B	2.88 (1.67–4.22)	0.35 (0.14–0.79)	0.36 (0.17–1.56)	0.22		
		Group C	0.69 (0.27–2.44)	0.46 (0.22–1.1)	0.24 (0.14–0.38)	0.55		
6	IL-6 (pg/ml)	Group A	13.88 (5.83–36.54)	14.52 (5.09–33.29)	22.29 (5.98–105.94)	0.78	0.271	0.916
		Group B	8.13 (3.81–12.2)	11.21 (4.58–31.67)	11.69 (6.67–54.23)	0.23		
		Group C	17.17 (3.88–39.73)	8.87 (4.96–22.95)	21.33 (7.81–32.38)	0.55		
7	IL-7 (pg/ml)	Group A	6.16 (2.81–10.3)	4.47 (2.77–10.23)	3.39 (2.19–9.66)	0.24	0.311	0.603
		Group B	3.75 (2.29–5.24)	2.7 (1.78–5.41)	3.68 (1.69–5.08)	0.69		
		Group C	3.78 (2.08–6.06)	4.74 (2.49–8.48)	3.71 (1.79–5.98)	0.1		
8	IL-8 (pg/ml)	Group A	1589.84 (94.64–2814.19)	1290.48 (105.63–3925.97)	1648.81 (522.78–4837.69)	0.73	0.118	0.841
		Group B	111.9 (37.5–1671.22)	1247.77 (445.95–1964.53)	1141.64 (286.89–3743.95)	0.33		
		Group C	2331.61 (87.84–3645.23)	543.09 (141.76–1350.67)	2141.51 (366.62–3505.66)	0.39		
9	IL-13 (pg/ml)	Group A	11.25 (5.21–18.36)	18.03 (8.51–35.56)	16.87 (10.34–49.96)	0.79	0.28	0.714
		Group B	16.87 (8.51–29.32)	15.69 (3.36–45.12)	13.93 (6.92–54.09)	0.16		
		Group C	20.79 (9.77–46.52)	24.29 (16.56–40.81)	13.93 (9.43–40)	0.44		
10	IL-18 (pg/ml)	Group A	76.84 (55.65–112.39)	74.06 (43.97–115.96)	64.29 (50.38–116.55)	0.26	0.0846	0.901
		Group B	65.91 (53.12–89.69)	77.68 (62.18–104.55)	70.48 (55.82–118.18)	0.62		
		Group C	54.86 (43.24–82.09)	55.1 (44.32–78.36)	53.22 (41.92–112.45)	0.39		
11	IP-10 (pg/ml)	Group A	397.39 (226.52–509.64)	318.07 (108.19–419.83)	327.57 (145.22–453.67)	0.58	0.506	0.158
		Group B	253.98 (187.69–426)	252.32 (200.42–423.24)	309.83 (203.02–367.4)	0.84		
		Group C	311.58 (203.43–473.01)	273.6 (143.32–392.26)	181.46 (41.8–276.19)	0.13		
12	MCP-1 (pg/ml)	Group A	865.52 (592.75–1244.08)	1007.43 (459.96–1541.01)	832.16 (463.76–1169.22)	0.9	0.0258,^b^	0.515
		Group B	841.79 (571.82–1041.41)	728.13 (508.48–1432.78)	1228.18 (669.23–1970.4)	0.43		
		Group C	1226.77 (810.12–1849.29)	928.61 (740.18–1035.01)	930.41 (790.17–1392.61)	0.29		
13	MCP-3 (pg/ml)	Group A	89.35 (12.62–215.72)	78.44 (13.82–230.03)	152.67 (44.16–318.96)	0.64	0.0771	0.116
		Group B	44.23 (25.4–155.4)	58.89 (39.24–118.94)	59.22 (27.09–204.47)	0.79		
		Group C	166.85 (92.77–298.22)	33.41 (19.8–72.81)	47.93 (15.59–94.81)	0.0087,^##^		
14	TNF-α (pg/ml)	Group A	41.18 (26.32–59.9)	45.35 (21.05–75.4)	51.43 (27.54–64.03)	0.47	0.306	0.54
		Group B	25.52 (19.21–53.71)	40.38 (25.2–92.53)	49.28 (26.52–99.21)	0.003,^*,#^		
		Group C	37.46 (24.89–69.48)	36.74 (25.66–62.7)	35.1 (27.19–50.6)	0.38		
15	VEGFA (pg/ml)	Group A	480.99 (285.89–636.79)	377.62 (219.15–635.01)	381.35 (281.06–570.29)	0.01	0.126	0.495
		Group B	282.27 (162.7–493.66)	353.34 (258.11–538.16)	454.78 (225.08–600.5)	0.69		
		Group C	304.4 (184.82–473.7)	312.26 (208–491.95)	368.2 (188.75–442.4)	0.95		
16	HIF-1α (pg/ml)	Group A	164.24 (130.91–315.45)	193.6 (117.01–260.42)	140.03 (89.96–235.4)	0.036,^##^	0.4406	0.8979
		Group B	151.05 (119.55–253.94)	142.77 (94.96–184.16)	159.57 (95.94–228.1)	0.26		
		Group C	149.04 (109.67–199.79)	132.13 (96.34–226.47)	132.73 (91.4–228.36)	0.058		
17	vWF (ng/ml)	Group A	11.85 (3.35–23.61)	8.9 (4.74–14.2)	12.93 (5.32–21.85)	0.7538	0.5913	0.2296
		Group B	26.74 (12.1–30.9)	21.92 (18.74–24.51)	4.11 (3.03–7.3)	0.1353		
		Group C	17.3 (8.24–32.68)	17.31 (12.97–18.4)	10.35 (4.74–29.01)	0.8825		
18	hsCRP (mg/l)	Group A	5.85 (3.4–9.52)	3.76 (2.82–6.8)	6.8 (3–7.02)	0.186	0.705	0.0713
		Group B	6.8 (3.53–11.96)	5.15 (2.25–6.8)	4.2 (2.62–6)	0.093,^*^		
		Group C	6.8 (3.4–6.8)	6.8 (1.69–6.8)	3.4 (1.3–6.8)	0.257		
19	Serum ferritin (ng/ml)	Group A	98.4 (65.93–164)	105 (42.88–164)	75.6 (29.88–141)	0.009,^#^	0.735	0.963
		Group B	82 (36.55–155.5)	89.7 (25.98–137.25)	90.02 (29.75–181.5)	0.448		
		Group C	85.75 (45.78–140)	89.3 (52.35–140.25)	86.5 (36.03–152)	0.386		
20	D-dimer (ng/ml)	Group A	349 (277.5–442)	288 (245.25–423.25)	309.5 (236.5–420.5)	0.546	0.678	0.806
		Group B	292 (246–470)	356 (256–432)	378.5 (258.25–442.5)	0.753		
		Group C	407 (295–472.75)	341 (282.75–485.5)	329.5 (276.75–426)	0.061		

Values are expressed as median (interquartile range)

HIF-1α, hypoxia-inducible factor-1 alpha; hsCRP, high-sensitivity C-reactive protein; IFN-α2, interferon alpha-2; IFN-γ, interferon-gamma; IL, interleukin; IL-1Ra, interleukin-1 receptor antagonist; IP-10, interferon γ-induced protein 10 kDa; MCP, monocyte chemoattractant protein; ng/ml, nanogram per millimeters; pg/ml, picograms per millimeters; TNF-α, tumor necrosis factor alpha; VEGFA, vascular endothelial growth factor A; vWF, von Willebrand factor

^*^Before treatment vs. mid-treatment; ^#^, before treatment vs. after treatment; ^$^, mid-treatment vs. after treatment; ^b^, Group B vs Group C; * *p* < 0.05; ** *p* < 0.01; *** *p* < 0.001; **** *p* < 0.0001; * ∋ {*, #, $, b}

In Group B (treated with *Guduchi*) (*n* = 19) hematological parameters such as CBC, ESR, and PT/INR were observed in the normal range and remained within the normal range even after treatment. A slight increase in NMR was observed (Additional file 1). As regards, liver enzymes such as SGOT, and SGPT were slightly on the higher side at baseline and remained nearly the same after the treatment. Serum bilirubin (total bilirubin, and direct bilirubin) levels increased significantly although remained within normal limits till the end of the treatment. Kidney function parameters such as blood urea, serum creatinine, uric acid, total protein, albumin, and globulin were normal at baseline and remained so even after treatment. Serum creatinine levels significantly increased albeit within the normal range. The lipid profile was nearly within the normal limit and remained unchanged. We also observed that the median hsCRP significantly decreased from baseline 6.8 to 5.15 to 4.2 mg/l at the end of the treatment. Change in D-dimer and serum ferritin was not statistically significant in Group B. In Group C hematological parameters such as CBC, ESR, and PT/INR were observed in the normal range at baseline and remained within the normal range even after treatment. However, marginal decrease in hemoglobin and hematocrit values such as TRBC and HCT were observed to be statistically significant. Similarly, we observed a significant rise in eosinophil levels in Group C (Additional file 1) but well within normal limits. Liver functions parameters SGOT, SGPT, ALP, and bilirubin (Total, direct, and indirect) were within the normal limit and remained in the normal range except for ALP levels which decreased significantly. Kidney function parameters blood urea, creatinine, and uric acid remained unchanged. Total protein and globulin levels were significantly decreased at the end of the trial although within normal limits. The lipid profile was nearly at the normal limit and remained unchanged. There was no significant change observed in Group C with respect to COVID-19 disease activity specific parameters such as hsCRP, D-dimer, and serum ferritin. Although levels of D-dimer and hsCRP were lowered during the treatment, they were not statistically significant (Table 2, and Fig. 4).

#### Pro-inflammatory and anti-inflammatory cytokines

A total of 15 pro-inflammatory and anti-inflammatory cytokines levels were examined using their serum samples. The cytokine levels in Group A (*n* = 28) *(treated with Vasa)* did not change significantly except for VEGFA levels, which decreased significantly during the treatment (Table 2).

On the other hand, in Group B (*n* = 19) (treated with *Guduchi*) the level of TNF-α were observed significantly increased during the trial period (Table 2). Changes in all other cytokines were not statistically significant.

In Group C (*n* = 20) *(*combination of *Vasa* + *Guduchi)*, decreased MCP-3 while increased IL-1β levels were observed. IL-8 levels decreased from baseline, but after treatment, it restored to the baseline level. The values of some cytokine were below the detectable range in many samples such as IL-4 and IFN-γ (Table 2).

#### Molecular biomarkers for hypoxia and thrombosis

The levels of hypoxia and thrombosis biomarkers, HIF-1α and vWF were tested. The level of HIF-1α significantly decreased in Group A after treatment (AT) (Table 2 and Fig. 4G). Overall, the median of HIF-1α levels at baseline was similar in all groups; however, it would be worthwhile to highlight that there were few individuals with very high values in Group A, and their levels were lowered after treatment and came closer to the median. No significant change in HIF-1A levels in Group B and C was observed.

vWF, a molecular marker of thrombosis induced by HIF-1α, was undetectable in a large number of patients either before and/or after the treatment. The samples included in the analysis were Group A, *n* = 12; Group B, *n* = 6; Group B, *n* = 8. A decrease in the levels of vWF was observed in all three groups although not statistically significant. Overall, Group A showed a lowering effect on hypoxia and thrombosis markers HIF-1α, VEGFA (Table 2 and Fig. 4G, I) and PT/INR (Additional file 1).

Furthermore, to see the difference between groups we conducted a comparative analysis of the biochemical, hematological parameters, and molecular markers at baseline (referred to as "Before Treatment") and After Treatment. We observed that MCP-1 was significantly higher in Group C as compared to Group B at baseline (Table 2 and Additional file 1). However, we did not find any significant differences between groups in any other examined parameters.

### Management of complication

One patient from Group A and another from Group B with diabetes were on oxygen supplementation for 4 days since their condition deteriorated from mild to moderate stage, antipyretic, and antibiotics were also given along with intervention drugs. One of them continued with the trial medication and hence included in the analysis, whereas the other discontinued the medication and was considered a dropout. However, both were followed up till recovery and tested negative for RT-PCR during the trial period only.

## Discussion

The majority of SARS-CoV-2 infections had either no or mild symptoms. But there is also a significant proportion of infected individuals who suffer from a severe hyper-inflammatory and hypoxic response that entails the development of a potent and effective treatment for COVID-19. The result of this clinical trial demonstrated that the early-onset of oral treatment with *Vasa* (Group A), and *Guduchi* (Group B) with a dosage of 500 mg each, and a combination of these two drugs (Group C) with a dosage of 250 mg each twice a day, effectively prevented progression of COVID-19 disease from mild to moderate and/or severe state and was shown to be beneficial in the recovery process.

In our study, the average viral clearance observed was 13.92 days (95% CI 12.85–14.99) in Group A, 13.44 days (95% CI 12.14–14.74) in Group B and 11.86 days (95% CI 10.62–13.11) days in Group C. Furthermore, we also observed the median time taken to normal body temperature was 4 days in Group A and C, and 3 days in Group C. On the 7th day, 88% of patients in Group A and 90% of patients in Groups B and C had achieved normal temperature below 98.6 °F after treatment. This observation is comparable with another study in which avifavir, an RNA polymerase inhibitor, the treated group had a median time of two days to normalize body temperature and 4 days in the standard of care group [29]. Studies have reported a median viral clearance time to be 13 days after a 10-day treatment of hydroxychloroquine (HCQ) administration regardless of the severity of the sickness [30]. Another study that involved 396 non-severe COVID-19 patients found that the median time for viral shedding in the asymptomatic group was 14.5 days in contrast to 18.0 days in the symptomatic group [31]. Since our study does not involve any standard treatment control group, we compared it with the published data. Among the clinical symptoms, we observed improvement in fever, sore throat, headache, loss of smell, and loss of taste after treatment. Very few patients reported the persistence of some symptoms at the end of the intervention period, that too with lower grades. According to WHO Clinical Progression Scale [32], all the patients recruited in the study were mild (scores ≤ 2). During the clinical trial, two patients progressed to the moderate stage. At the end of the trial, all the patients in each group reverted to score of zero, i.e., healthy, asymptomatic, and uninfected.

Daily monitoring of oxygen saturation (SpO_2_) and respiratory rate revealed that none of the patients clinically progressed to higher stages or developed any complications. The heart rate, systolic, and diastolic blood pressure of patients remained normal throughout the trial period signifying the protective effect of *Vasa* and *Guduchi* on the cardiovascular system health during COVID-19. The leaves of AV are administered orally for bronchial asthma, colds, cough, whooping cough, dyspnea, fever, headache, phthisis, jaundice, and diarrhea [14]. The decoction prepared from *Adhatoda* leaves possesses a soothing effect that helps to clear throat irritation and can also act as an expectorant [33].

In addition to the clinical symptoms and viral clearance, we wanted to test the effect of *Vasa*, *Guduchi*, and a combination of *Vasa* and *Guduchi* on COVID-19-associated progression markers such as hsCRP, D-dimer, serum ferritin as well as inflammatory cytokines. We overall observed a decline in hsCRP in Group B and C, and serum ferritin in Group A. D-dimer levels were lowered in Group A and C but not statistically significant. In COVID-19, a high serum ferritin level is linked to worse outcomes and more severe illness, [34] and in the current study, we noted that the median ferritin level decreased from baseline 98.4 (65.93–164) to 75.6 (29.88–141) (ng/ml) at the end of the *Vasa* treatment in Group A. Among the inflammatory cytokines IP-10 decreased in Group A and C, whereas MCP-1 decreased significantly in Group C although before Bonferroni correction and MCP-3 remained statistically significant even after Bonferroni correction in Group C. Chen et al. have shown that critically ill patients had considerably greater serum IP-10, MCP-1, and MCP-3 levels than severe patients [35, 36]. In this study, AV treatment exhibited a beneficial effect on IP-10 and a combination of AV and TC reduced MCP-1 and MCP-3 levels. TNF-α was slightly increased in Group B.

SARS-CoV-2 is a systemic disorder that involved multiorgan effects to some extent. To estimate the overall safety of these drugs, we evaluated systemic health parameters such as CBC, ESR, PT, PT/INR, liver function, kidney function, and lipid profile. In our patient’s group, most parameters were within normal limits and were largely unaffected or altered within normal limits in all three groups. Almost half of SARS-CoV-2 infected individuals showed some impairment in their liver function and elevated liver enzymes [37]. Interestingly we observed a decrease in SGOT in Group A during the trial period which signifies that *Vasa* seemed to be effective in attenuating liver injury caused by the virus and other conditions. Patients with severe forms of COVID-19 had greater levels of bilirubin than those with milder ones [38]. In Group B, treatment did not exhibit any effect on slightly increased liver enzymes; instead, we observed a marginal increase in bilirubin levels that are reported to be raised in general in COVID-19 patients. Our results are in the line with a recent study in which TC was used as a part of the AYURAKSHA kit [39]. Group C also showed a decrease in ALP, serum protein, and globulin. However, in the middle of the treatment a decrease in Hb, TRBC, and HCT levels were observed although within the normal range. Hb and HCT values were within normal range and were not affected during the course of treatment in Group A and B.

The overall decline in the systemic health parameters has been observed and reported in literature during the course of the disease [40]. In a cohort of 127 patients with COVID-19, monocytosis (51.97%), lymphocytopenia (25.20%), eosinopenia (37.80%), and anemia (51.97%) were the most prevalent hematologic abnormalities observed [40]. Qin et al. also reported based on data from 452 patients that more severe cases had lymphocytopenia and greater leukocyte counts and lower percentages of monocytes, basophils, and eosinophils [41]. Overall, lung involvement, oxygen demand, and disease activity are related to the levels of alterations in leukocytes, neutrophils, lymphocytes, monocytes, eosinophils, basophils, and platelets as well as Hb, MCV, and MCHC. Furthermore, eosinophils of moderate patients recovered faster than those of severe, suggesting that dynamical eosinophils may be the key to COVID-19 recovery [40, 42]. During our study, we observed eosinophil levels considerably increased in both A and C Groups. It is also reported that eosinophils also play a crucial role in viral infections, such as HIV infection and respiratory syncytial virus [43, 44]. The monocyte levels were decreased from baseline to the end of the *Vasa* treatment. A meta-analysis by Ulloque-Badaracco et al. reported that a total of 11,356 patients from 31 cohort studies had lower AGR in severe COVID-19 and non-survivors than non-severe COVID-19 patients [45, 46]. Our clinical trial findings also suggest that *Vasa* and combination of *Vasa* and *Guduchi* treated groups showed an increase in AG ratio and subsequently decreased Globulin levels.

It is reported that without any indications of respiratory discomfort or dyspnea, patients with COVID-19 are frequently shown to have life-threatening hypoxemia which is termed as Silent hypoxia. The viral ORF3a protein is shown to increase the production of HIF-1α during SARS-CoV-2 infection, which in turn promotes the inflammatory responses and subsequently the infection. Since HIF-1α is a critical promoter of both SARS-CoV-2 infection and inflammatory response, it is suggested as a viable therapeutic target for COVID-19 and virus-induced inflammatory infection [47]. Since COVID-19 hypoxemia is linked to a higher risk of death, early detection and rapid treatment are crucial to avert any complications [48]. In the current study, hypoxia markers were investigated to understand the effect of trial drugs on them, and a variability in the levels of HIF-1α at baseline were observed. A significant decrease in HIF-1α levels was detected during the trial after treatment with *Vasa* in Group A. Our earlier preclinical study on AV by Gheware et al. showed that in mouse models of hypoxia-hemostasis, pulmonary fibrosis, and sepsis, in addition to other parameters, HIF-1α levels were reduced with oral treatment of AV [18]. Another interesting aspect of SARS-CoV-2 infection is “hypoxithrombosis” [49]. Elegant studies have shown a significant increase in vWF antigen and its activity in patients with COVID-19, which could be a factor in the higher risk of thrombosis [50]. In our study, a large number of patients had undetectable values of vWF before and/or after the treatment because it was conducted on mildly symptomatic patients. In Group B we observed decreased of vWF levels although not statistically significant. Further, VEGFA levels significantly lowered in Group A. In Group A, we also observed PT/INR significantly increased within normal limits which is also an indicator of AV having preventive effects on thrombotic outcomes of hypoxia and COVID-19. In mice, it is shown that AV extract reverse the inflammatory and blood coagulation characteristics [18].

Although this analysis provided in-depth understanding of the clinical effects of tested formulation in COVID-19 patients, it has two limitations. One, due to the highly contagious nature of the disease, the patient's willingness to comply with blood sample collections was adversely affected. As a result, this left us with less than half of the patients from whom three-timepoint sample workups. Second, the study did not include a placebo control group. In the context of the COVID-19 pandemic, the absence of established therapeutic interventions necessitated the implementation of symptomatic management as the prevailing standard of care. The Government of India, Ministry of AYUSH, subsequently issued guidelines for the management of COVID-19, incorporating Ayurvedic medicines, with *Tinospora cordifolia* extract serving as a designated Ayurvedic standard of care intervention for mild cases. In this study, we adopted this Ayurvedic approach as one arm of our investigation, while *Adhatoda vasica* was considered as another distinct arm. Additionally, our research aimed to assess the potential synergistic effects arising from the concurrent administration of both Ayurvedic drugs to determine if any supplementary benefits could be observed.

This study demonstrated the effects of *Vasa ghan*, *Guduchi ghan,* and a combination of *Vasa* and *Guduchi Ghan* on mild COVID-19 patients. This is the first clinical trial study based on preclinical leads involving traditional Indian Ayurvedic medicine treatment with effects observed on multiple clinical, biochemical, and molecular parameters at different timepoints.

## Conclusions

Even though the alarmingly high number of SARS-CoV-2 cases are asymptomatic or mild, they can also manifest as severe viral pneumonia with hyper-inflammatory response, demanding the use of powerful and effective medication. We present the first clinical evidence of HIF-1 reduction in COVID-19 patients receiving AV herbal extract treatment. Our study concluded that the use of oral medication of *Vasa* ghan, *Guduchi* ghan, and *Vasa Guduchi* ghan are effective treatment for mild COVID-19 patients. All three treatment arms prevented the disease’s progression to its more severe stages. Immunomodulatory activity of *Guduchi*, anti-inflammatory, anti-hypoxic, and anti-thrombotic effects observed for *Adhatoda* at preclinical levels were also seen in the clinical study. This is also the first report of in-depth analysis of clinical outcomes of Ayurvedic interventions on COVID-19 mild cases, assessed through multiple parameters analyzed at different time points. Taken together, the results of viral clearance and other systemic beneficial outcomes of host response established the potential of AV, TC, and AV + TC to be safe and efficacious interventions for COVID-19 at doses tested.

### Supplementary Information

**Additional file 1.** Lab technical and administrative support.

## Data Availability

The data sets used and analyzed during the current study are available from the corresponding author upon reasonable request.

## Abbreviations

A/G ratio: Albumin/globulin ratio
ALP: Alkaline phosphatase
API: Ayurvedic Pharmacopoeia of India
AV: *Adhatoda vasica*
COVID-19: Coronavirus disease-2019
ESR: Erythrocyte sedimentation rate
Hb: Hemoglobin
HCT: Hematocrit
HIF-1α: Hypoxia inducible factor-1 alpha
hsCRP: High-sensitivity C-reactive protein
HDL: High-density lipoprotein
IFN-α2: Interferon alpha-2
IFN-γ: Interferon-gamma
IL: Interleukin
IL-1Ra: Interleukin-1 receptor antagonist
INR: International normalized ratio
IP-10: Interferon γ-induced protein 10 kDa
LNR: Lymphocyte-to-neutrophil ratio
MCH: Mean corpuscular hemoglobin
MCHC: Mean corpuscular hemoglobin concentration
MCP: Monocyte chemoattractant protein
MCV: Mean corpuscular volume
NMR: Neutrophil-to-monocyte ratio
PT: Prothrombin time
SARS-CoV-2: Severe acute respiratory syndrome coronavirus 2
SGOT: Serum glutamic oxaloacetic transaminase
SGPT: Serum glutamate pyruvate transaminase
TC: Tinospora cordifolia
TNF-α: Tumor necrosis factor alpha
TRBC: Total red blood cells
TLC: Total leukocytes count
VEGFA: Vascular endothelial growth factor A
VLDL: Very low-density lipoproteins
vWF: Von Willebrand factor
